# Influence of analytic methods, data sources, and repeated measurements on the population attributable fraction of lifestyle risk factors

**DOI:** 10.1007/s10654-023-01018-z

**Published:** 2023-06-06

**Authors:** You Wu, Hanseul Kim, Kai Wang, Mingyang Song, Molin Wang, Rulla Tamimi, Heather Eliassen, Stephanie A. Smith-Warner, Walter. C. Willett, Edward L. Giovannucci

**Affiliations:** 1 Institute for Hospital Management, School of Medicine, Tsinghua University, Beijing, China; 2 Department of Health Policy and Management, Bloomberg School of Public Health, Johns Hopkins University, Baltimore, MD USA; 3 Department of Nutrition, Harvard T.H. Chan School of Public Health, Boston, MA USA; 4 Department of Biostatistics, Harvard T.H. Chan School of Public Health, Boston, MA USA; 5 Clinical and Translational Epidemiology Unit, Massachusetts General Hospital and Harvard Medical School, Boston, MA USA; 6 Division of Gastroenterology, Massachusetts General Hospital and Harvard Medical School, Boston, MA USA; 7 Department of Epidemiology, Harvard T.H. Chan School of Public Health, Boston, MA USA; 8 Channing Division of Network Medicine, Department of Medicine, Brigham and Women’s Hospital and Harvard Medical School, Boston, MA USA; 9 Department of Population Health Sciences, Weill Cornell Medicine, New York, NY USA

**Keywords:** Population attributable risk, Lifestyle, Breast cancer, Cancer preventability, Repeated measurements

## Abstract

Population attributable risk (PAR%) reflects the preventable fraction of disease. However, PAR% estimates of cancer have shown large variation across populations, methods, data sources, and timing of measurements. Three statistical methods to estimate PAR% were identified from a systematic literature review: the Levin’s formula, the comparative incidence rate method, and the comparative risk assessment method. We compared the variations in PAR% of postmenopausal breast cancer in the Nurses’ Health Study to evaluate the influence by method choice, source of prevalence data, use of single vs repeated exposure measurements, and potential joint effects of obesity, alcohol, physical activity, fruit and vegetable intake. Across models of the three methods, the estimated PAR% using repeated measurements were higher than that using baseline measurement; overall PAR% for the baseline, simple update, and cumulative average models were 13.8%, 21.1%, 18.6% by Levin’s formula; 13.7%, 28.0%, 31.2% by comparative risk assessment; and 17.4%, 25.2%, 29.3% by comparative incidence rate method. The estimated PAR% of the combination of multiple risk factors was higher than the product of the individual PAR%: 18.9% when assuming independence and 31.2% when considering the risk factors jointly. The three methods provided similar PAR% based on the same data source, timing of measurements, and target populations. However, sizable increases in the PAR% were observed for repeated measures over a single measure and for calculations based on achieving all recommendations jointly rather than individually.

**Supplementary Information:**

The online version contains supplementary material available at 10.1007/s10654-023-01018-z.

## Introduction

Cancer is a leading cause of disease burden and mortality across countries [1, 2], while a substantial proportion could be prevented by primary intervention [3]. The population attributable risk (PAR%) estimates the percentage of disease in a target population that would not have occurred through the optimization of its etiologic factors. As a causal parameter, PAR% only reflects the theoretically avertable cases if the given risk factors are completely eliminated in a population; it does not always translate into the actual proportion of disease that could be prevented. Its magnitude, however, has practical interpretation and informative value in prevention planning.

Various statistical methods exist to estimate the PAR% of disease incidence. *Levin’s formula* computes the product sum of the exposure prevalence from national or regional representative populations, and the relative risks from published literature [4]. It usually classifies the target population into high-risk and low-risk exposure categories, depending on the original survey data structure. The *comparative incidence rate* method compares the age-standardized incidence rate for the disease in a low-risk reference group to the rate in the entire study population, assuming that the low-risk group had fewer cases due to less exposure to the risk factors of interest, and the remaining characteristics compared to the total population are otherwise the same [3]. The *comparative risk assessment* estimates the PAR% of cancer by summing up the burden attributable to each stratum of the risk factor compared with the theoretical-minimum-risk exposure [5]. Differences in the underlying assumptions of each method may result in variability in the estimations they provide. For example, Levin's formula takes on a simpler form because it assumes the risk within each category is identical; whereas comparative risk assessment requires more computation resources to achieve an integral calculation with much finer strata.

For each method, the data sources for the prevalence and relative risks can also differ. To estimate the prevalence, some studies have used cross-sectional surveys [6–8], while others have used empirical data from cohort studies [9]. To estimate the relative risks, results have been extracted from the published literature, from observational studies with one exposure assessment [7], and from observational studies with repeated exposure measurements [9]. Results from studies based on a single measure are more prone to measurement error of long-term exposure than those using repeated measures; if the relative risk is underestimated, the PAR% likewise will be underestimated.

Besides the aforementioned discrepancies, studies have not been consistent in whether to combine the PAR% of individual risk factor under the independence assumption [10], or to model multiple factors jointly [11]. The consideration of joint distribution incorporates possible synergy, and if the relationship between the exposures and the disease is causal, the overall PAR% would represent the theoretically preventable fraction when all risk factors were at the optimal level simultaneously.

So far, no research has yet systematically summarized the impact on the PAR% by use of the different methods, the data sources, and/or consideration of risk factors individually or jointly in estimating the risks and prevalence. The magnitude of the preventable fraction of breast cancer, for example, has varied greatly across studies even if the same or similar risk factors were considered. Therefore, we systematically reviewed the original studies on PAR% of breast cancer by modifiable lifestyle risk factors, and then evaluated in the Nurses’ Health Study (NHS) the degree to which PAR% of postmenopausal breast cancer varied (1) by three statistical methods; (2) by using single versus repeated exposure measurements to estimate the relative risks; (3) by the source of the prevalence data; and (4) by considering exposures individually and then summing the estimates or assuming optimal risk in individuals who achieve all the low-risk behaviors. We calculated the PAR% of four lifestyle risk factors in the 2018 World Cancer Research Fund and American Institute for Cancer Research (WCRF/AICR) cancer prevention recommendations for which the evidence for an association with breast cancer was strong (alcohol consumption, body mass index [BMI], and physical activity) or suggestive (fruit and vegetable intake) [12].

## Methods

### Systematic literature review

A systematic review was conducted by searching PubMed, Embase, and Web of Science up to 10/1/2022 to include original studies on PAR% of breast cancer by modifiable lifestyle risk factors. Studies were excluded if they were duplicates, were not original research articles, were not conducted in women, were not on breast cancer incidence, or evaluated non-modifiable risk factors of breast cancer. The search strategy is listed in Table S1 and the literature screening process is summarized in Figure S1.

### Nurses’ Health Study

The NHS was established in 1976 when 121,701 female nurses aged 30–55 years returned the initial questionnaire [13]. Participants have been followed up biennially to collect their medical, lifestyle, and other health-related information. The overall response rate has achieved 85%-90% in most follow-up cycles of the questionnaires [14].

#### Assessment of exposures

In 1980, 1984, 1986 and every 4 years thereafter, participants returned semiquantitative food frequency questionnaires (FFQ) covering their usual diet in the past year. Fruit and vegetable consumption was estimated based on the quantity and frequency of all relevant food items consumed. Alcohol intake was estimated based on the alcohol content of the alcoholic beverages consumed [15, 16]. Moderate to high validity (Supplementary methods) of the FFQ in measuring intake of alcohol and fruit and vegetables has been documented [17, 18]. Starting in 1986 and every 2–4 years thereafter, participants reported their average time per week spent engaging in various types of physical activity [19]. Total physical activity was converted into metabolic equivalent task hours per week [20]. Information on height was collected in 1976. Weight was reported in 1976 and biennially afterwards [21]. Dairy and calcium intakes were considered as suggestive risk factors by WCRF/AICR, but were not included based on the null result from the latest comprehensive assessment in a large international consortium of diet and breast cancer [22], which was published after the latest WCRF/AICR recommendations.

For each of the methods, the exposures were modeled in three ways. The baseline exposures were used as fixed variable. The repeat assessments were included as simple update values, or used to compute the cumulative average exposures. For instance, the incidence of breast cancer from 1994 to 1996 was associated with the lifestyle factors measured in 1994 in the simple update model; whereas in the cumulative average model, the incidence from 1994 to 1996 was associated with the averaged exposure from 1986 to 1994. An illustration explaining the modeling details is visualized in Figure S2.

#### Assessment of covariates

Age at menarche was collected in 1976. Menopausal status and history of benign breast disease were assessed at baseline and updated biennially. Family history of breast cancer was obtained in 1982 and updated every 4 years beginning in 1988. Oral contraceptive use was assessed in 1980, 1982, and 1984. Age at first birth was asked updated biennially until 1982. Parity was asked biennially until 1996. Menopausal hormone therapy (MHT) use was asked biennially until 2004.

#### Case ascertainment in NHS

Participants in the NHS were asked to report the diagnosis of invasive breast cancer biennially. Medical records and pathology reports were retrieved upon participant permission (or next of kin for those who had died) to confirm the identified diagnosis. Only postmenopausal breast cancer cases were included in the analysis.

### US national exposure prevalence data

US national distributions for alcohol consumption were obtained from the National Health Interview Survey (NHIS, 2013 and 2014) [23]. Information on BMI, fruit and vegetable intake and physical activity were obtained from the National Health and Nutrition Examination Survey (NHANES, 2011-2012, 2013-2014) [24].

### Risk estimates for lifestyle factor and breast cancer associations from meta-analyses

Relative risk (RR) estimates for each risk factor were identified from the most updated meta-analysis on their association with risk of breast cancer incidence. When the cut points in the meta-analysis did not match the national surveys, we imposed a log-linear relationship on the RR from continuous analysis and calculated the RR for each level of the exposure.

### Statistical analysis

Year 1986, when physical activity was first asked in the NHS, was considered as the baseline. Participants were excluded if they had a history of cancer (except for nonmelanoma skin cancer), missing value for any of the four main exposures (alcohol, BMI, fruits and vegetables, and physical activity) at baseline, or had extreme total energy intake (below 600 or above 3500 kcal/day).

We calculated RRs of postmenopausal breast cancer in which each exposure was modeled as binary variables representing high- (listed first) and low-risk categories based on the WCRF/AICR cancer prevention recommendations [25]: alcohol consumption (drinker vs non-drinker), BMI (≥ 25 vs < 25 kg/m^2^), fruit and vegetable intake (< 5 vs ≥ 5 servings/day), and physical activity (< 18 vs ≥ 18 MET-hours/week). The value from the last questionnaire returned was carried forward when missing. Age and multivariable-adjusted relative risks (and 95% confidence intervals [CI]) were estimated using Cox proportional hazards models. For models using repeated measurements, exposures up to the assessment just before diagnosis, loss to follow-up, or the last assessment before the end of follow-up were used, and the covariates were included as time-varying variables whenever possible. Specific categorizations of the covariates included in the multivariable models can be found in Table 4.

#### Levin’s formula

For each risk factor when classified as a categorical variable, we applied the modified Levin’s formula by Hanley J. to accommodate the multi-level risk factors [26]:

$$Individual\,PAR\%= \frac{\sum_{n=1}^{N}{P}_{n}*\left(R{R}_{n}-1\right)}{\sum_{n=1}^{N}{P}_{n}*\left(R{R}_{n}-1\right)+1 }$$

where *RR*_*n*_ is the relative risk associated with the *n-th* level of a given risk factor, *P*_*n*_ is the prevalence of the *n-th* high risk category of the risk factor in the study population, and N is the total number of categories.

The overall PAR% was then computed by applying the multiplicative formula, under the assumption of independent exposures and effects under study [10]:

$$Overall\,PAR\%=1- \prod_{k=1}^{K}(1-PA{R}_{k}\%)$$

where *PAR*_*k*_% denotes the PAR% of the *k*-th risk factor. K is the total number of risk factors.

#### Comparative incidence rate method

Also known as the low-risk method, this model firstly defines a low-risk reference group as those being at the optimal level for all risk factors. Age-specific postmenopausal breast cancer incidence rates were calculated for the low-risk group in NHS and the entire NHS cohort in 5-year age groups, then standardized by the number of people in each age group. PAR% (Model 1) was calculated as:

$$PAR\% = \frac{{({\text{ASIR}} _{NHS} {-} {\text{ASIR}}_{low{\text{-}} risk} ) }}{{{\text{ASIR}}_{NHS} }}$$

where ASIR_NHS_ is the age-standardized incidence rate in NHS, ASIR_low-risk_ is the age-standardized incidence rate in the low-risk group.

We calculated the PAR% in the US population as:

$$PAR\% = \frac{{\left( {{\text{ASIR }}_{US {\text{-}} 55 + } {-}{\text{ ASIR}}_{low {\text{-}} risk} } \right){ }}}{{{\text{ASIR}}_{US {\text{-}} 55 + } }}$$

where ASIR_US-55+_ is the US age-standardized incidence rate among women over 55-year-old, at which age over 90% of US women would have become postmenopausal [27, 28], and ASIR_low-risk_ is the age-standardized incidence rate in the low-risk group.

In sensitivity analyses, we limited the study population to non-users of MHT, which may modify he effect of BMI, including never users and those who took MHT for less than 10 years and stopped using it for four years or longer [29].

#### Comparative risk assessment

The comparative risk assessment firstly obtains the joint classification of the four risk factors, estimates the excess risk compared with the theoretical-minimum-risk exposure that could be attributable to each stratum of the multi-factorial combination, and then calculates the integral of the PAR% in possible levels of the combinations:

$$PAR\% = \frac{{\mathop \smallint \nolimits_{x = 0}^{m} RR\left( x \right)P\left( x \right)dx - \mathop \smallint \nolimits_{x = 0}^{m} RR\left( x \right)P^{\prime } \left( x \right)dx}}{{\mathop \smallint \nolimits_{x = 0}^{m} RR\left( x \right)P\left( x \right)dx}}$$

where x represents each exposure level of the joint classification, P(x) represents the distribution of the stratum, P′(x) represents the distribution of the optimal stratum, RR(x) the relative risk of a given stratum compared to the reference (i.e., the optimal stratum), and m the total number of possible combinations of the risk factors.

A partial PAR% was estimated as the proportional reduction expected in disease incidence if all the risk factors of interest were set to the optimal level while holding the other covariates unchanged. We calculated partial PAR% and 95% CIs using the %PAR SAS macro by Spiegelman et al. [30]. We estimated the overall PAR% by two approaches. One classified the participants into the high- and the low-risk groups based on the joint distribution of the four risk factors; the other took in the four risk factors individually into the model to obtain a composite partial PAR% for being at elevated risk for each of the factors [30].

### Sensitivity analysis

In sensitivity analyses, we substituted BMI with adult weight change to examine the PAR%. We also corrected for fruit and vegetable intake relative to dietary records [17] to evaluate the bias due to over-reporting.

For all hypothesis tests, a *p-value* < 0.05 was considered statistically significant, and all tests of statistical significance were 2-sided. Analyses applying Levin’s formula were conducted using R, version 3.6.0 (R Foundation for Statistical Computing, Vienna, Austria). All other analyses were conducted using SAS, version 9.4 (SAS Institute, Inc., Cary, NC).

## Results

### Systematic literature review

A total of 238 studies went through abstract review. After excluding non-research articles, molecular or animal studies, and studies on breast cancer survival, 99 went through full-text review. Further excluded were the studies of non-modifiable risk factors, or if no specific PAR% was presented. A total of 62 publications on PAR% of breast cancer by modifiable lifestyle risk factors were identified (8, 9, 11, 33, 35, 37–92). Figure S3 summarizes the region, source of relative risk (RR), statistical method, and the risk factors evaluated for the 62 studies. Specific study characteristics are listed in Table S2. The majority of the studies were of American/European origin (n = 41). The relative risk information of nearly 70% of the studies came from meta-analyses (by author's literature search or the WCRF Continuous Update Report), whereas the rest were obtained from the specific case–control studies or cohort studies (2 studies [3, 9] were based on the same population of our study). Thirty-six studies applied Levin/Hanley’s formula, 11 used the comparative incidence rate method, and 11 employed comparative risk assessment. Alcohol, obesity, physical activity, and diet were the four most common risk factors studied. As summarized in Table 1, 21 studies reported PAR% for multiple risk factors, among which 10 did not report the overall PAR% or the specific method used; 7 assumed independent effects, while 4 considered the factors jointly. Substantial variability in PAR% was observed even in studies of single factors. Among 3 studies that considered alcohol, BMI and physical activity, the PAR% varied from 11.9 to 30.0%.

**Table 1:** Summary of studies on population attributable risk (PAR%) of breast cancer by modifiable lifestyle risk factors

Lifestyle risk factors						Number of studies	Range of PAR%
*Single factor considered individually*							
Alcohol	BMI	Physical activity	Weight gain	Smoking	Diet		
●						20	0.2%–20.1% (median: 5.1%)
	●					39	1.0%–49.3% (median: 12.0%)
		●				28	0.6%–28.8% (median: 7.0%)
			●			3	16.8%–21.3% (median: 18.7%)
				●		4	3.1%–11.9% (median: 4.6%)
					●	8	3.2%–11.5% for food/nutrients 18.0%–20.0% for dietary pattern
*Multiple factors considered jointly*							
Alcohol	BMI	Physical activity	Weight gain	Smoking	Diet		
●	●					1	17.4%
●		●				1	12.4%
●	●	●				3	11.9%–30.0%
●		●	●			1	37.7%
●	●	●		●		2	15.0%–19.4%
●	●	●		●	●	1	25.7%
●		●		●		1	21.4%
	●	●				1	40.7%

### Cohort information

In our study of the NHS population, compared with the high-risk group, women in the low-risk group consumed no alcohol, had lower BMI, higher fruit and vegetable intake, and higher physical activity at baseline. The mean age, height, and age at menarche, distribution of parity and age at first birth, the proportion with a personal history of benign breast disease or family history of breast cancer, and menopausal status/MHT use were similar between the groups. Women in the low-risk group were less likely to be current smokers (Table 2). Fewer women were classified as non-drinkers based on cumulative average intake over follow-up as compared to baseline. Throughout study follow-up, fruit and vegetable intake remained about the same, whereas BMI and total physical activity tended to increase (Table S3). We identified 6708 incident invasive postmenopausal breast cancer cases during 28 years of follow-up in the NHS.

**Table 2:** Baseline characteristics of participants according to high and low risk status of postmenopausal breast cancer^a^ in the Nurses' Health Study (n = 73,143)

	Low-risk group	High-risk group
	Mean (SD) or percentage (%)	
**Risk factors**		
Alcohol consumption, g/day	0 (0)	6.3 (10.8)
Body mass index (BMI), kg/m^2^	22.1 (1.8)	25.4 (4.8)
Fruit and vegetable intake, servings/d	7.7 (2.7)	4.9 (2.6)
Physical activity, MET-hrs^b^/week	40.3 (28.1)	13.5 (20.2)
**Covariates**		
Age, years	53.2 (7.4)	52.5 (7.2)
Height, m	1.64 (0.06)	1.64 (0.06)
Age at menarche, years	12.6 (1.8)	12.4 (1.8)
Race		
White	95.9%	97.8%
Parity and age at first birth (years)		
Nulliparous	6.5%	5.8%
1–2 children age at 1st birth < 25	14.8%	13.9%
1–2 children age at 1st birth 25–29	17.0%	15.3%
1–2 children age at 1st birth ≥ 30	6.9%	6.2%
3–4 children age at 1st birth < 25	23.1%	25.8%
3–4 children age at 1st birth 25–29	17.2%	16.3%
3–4 children age at 1st birth ≥ 30	2.4%	2.6%
≥ 5 children	7.1%	9.2%
Missing parity or age at 1st birth	5.0%	4.8%
Oral contraceptive (OC) use		
No OC use	59.2%	52.9%
> 0–2 years of OC use	15.7%	17.9%
2–5 years of OC use	11.3%	12.9%
> 5–10 years of OC use	10.1%	11.2%
> 10 years of OC use	3.7%	5.0%
History of benign breast disease	31.2%	29.8%
Family history of breast cancer	8.0%	8.1%
Menopausal hormone therapy (MHT) use		
Premenopausal/unknown menopausal status	30.7%	33.4%
Never user among postmenopausal women	33.8%	35.4%
Past user among postmenopausal women	15.8%	14.3%
Current user among postmenopausal women	19.6%	17.0%
Smoking status		
Never smoker	58.9%	43.8%
Past smoker	28.2%	34.9%
Current smoker	13.0%	21.3%

^a^Low-risk status for breast cancer defined by meeting the following four criteria: alcohol consumption (0 g/day), body mass index (< 25 kg/m^2^), fruit and vegetable intake (≥ 5 servings/day), and physical activity (≥ 18 total MET-hrs/week). Others were defined as having high risk

^b^MET-hrs, metabolic equivalent of task per hour, a measure of relative intensity of different physical activities as compared to the resting metabolic rate

### Parameters of PAR%: relative risks, prevalence, and incidence rate

In the NHS, higher alcohol consumption, higher BMI, and lower fruit and vegetable intake were associated with higher risk of postmenopausal breast cancer. Low physical activity was marginally associated with higher risk of postmenopausal breast cancer. The associations tended to be weaker in the baseline model compared to the other two methods using repeated measures (Table S4). The summary RRs and prevalences from meta-analyses [31–33] are listed in Table S5.

The prevalence data from NHS are shown in Table S4 and S6; the proportion of total person-time allocated to high-risk categories was generally higher in models with repeated measurements, except for physical activity. The distribution of the risk factors in the US population was obtained from NHANES and NHIS (Table S6). Table S7 shows the age-standardized incidence rate for postmenopausal breast cancer in the low-risk group in NHS, in all NHS participants, and in the general US population. The rate for the low-risk group ranged from 312.7 per 100,000 person-years (risk classification by baseline measurement) to 267.9 per 100,000 person-years (risk classification by cumulative average measurements). The incidence rate for the entire NHS cohort was slightly higher (378.8/100,000 person-years, 1986–2014) than that for the US general population (358.2/100,000 person-years, 1975–2017), probably due to differences in MHT use, reproductive variables, or breast cancer screening.

### PAR% estimates

#### Single vs repeated measurements

**Fig. 1:**
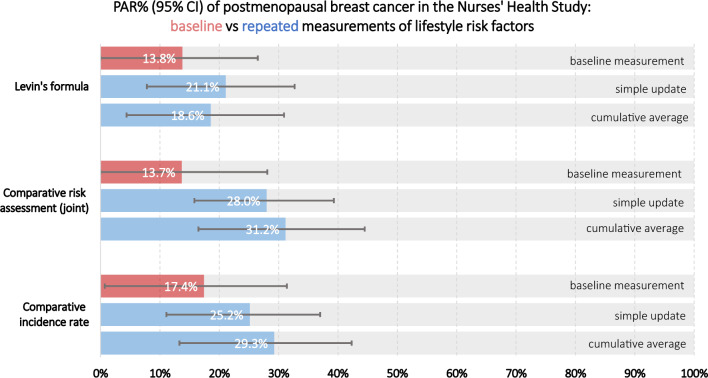
PAR% (95% CI) of postmenopausal breast cancer in the Nurses' Health Study: baseline vs repeated measurements of lifestyle risk factors

Table 3 shows the PAR% of postmenopausal breast cancer by each analytic method, source of relative risks, and prevalence and timing of exposure measurements. Across models of the three methods, the results are generally consistent given the same data source and timing of exposure measurement; the estimated PAR% using repeated measurements, however, was higher than that using baseline measurement (Fig. 1). Had everyone had their alcohol consumption, BMI, fruit and vegetable intake, and physical activity at the low-risk levels, the PAR% of postmenopausal breast cancer by the 4 lifestyle risk factors in NHS was 13.8% for the baseline model, 21.1% for the simple update model, and 18.6% for the cumulative average model as estimated by Levin’s formula. When applying comparative risk assessment, the estimated preventable fraction was 13.7% for the baseline model, 28.0% for the simple update model, and 31.2% for the cumulative average model, holding the other covariates unchanged. The PAR% in NHS estimated by comparative incidence rate method was 17.4% when using only baseline data, 25.2% for the simple updated model, and 29.3% for the cumulative averaged model.

**Table 3:** Population attributable risk (PAR%) of postmenopausal breast cancer by lifestyle risk factors^a^ according to the sources of relative risks, prevalence, and timing of exposure measurements

Method and target population		Source of relative risk	Source of prevalence	Timing of measurement	PAR%	95% CI
1	Levin's formula (NHS)	NHS	NHS	Baseline	13.8	(0%–26.5%)
				Simple update	21.1	(7.8%–32.7%)
				Cumulative average	18.6	(4.4%–30.9%)
2	Levin's formula (US)	NHS	NHANES & NHIS	Baseline	20.9	(1.9%–36.3%)
				Simple update	27.4	(10.4%–41.4%)
				Cumulative average	27.0	(7.8%–42.5%)
3	Levin's formula (US)	Meta-analysis	NHANES & NHIS	-	25.6	(17.3%–34.5%)
4	Comparative risk assessment (NHS, joint effect)	NHS	NHS	Baseline	13.7	(0%–28.1%)
				Simple update	28.0	(15.8%–39.3%)
				Cumulative average	31.2	(16.5%–44.5%)
5	Comparative risk assessment (NHS, individual)	NHS	NHS	Baseline	12.5	(1.6%–23.2%)
				Simple update	20.7	(10.7%–30.3%)
				Cumulative average	18.9	(7.9%–29.5%)
6	Comparative incidence rate (NHS)	Low-risk group in NHS compared to overall NHS		Baseline	17.4	(0.7%–31.4%)
				Simple update	25.2	(11.1%–37.0%)
				Cumulative average	29.3	(13.3%–42.3%)
7	Comparative incidence rate (US)	Low-risk group in NHS compared to US		Baseline	12.7	(0%–27.3%)
				Simple update	20.8	(5.0%–34.1%)
				Cumulative average	25.2	(10.2%–37.7%)

^a^Alcohol consumption, BMI, fruit and vegetable intake, and physical activity

#### Individual vs joint effect

**Fig. 2:**
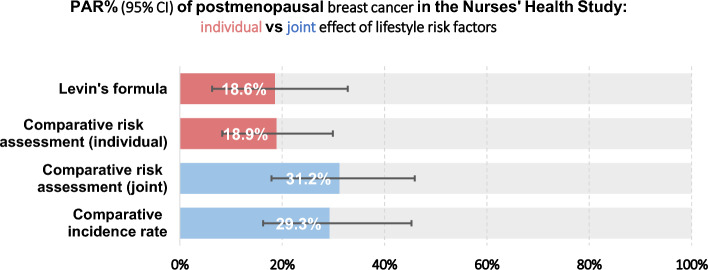
PAR% (95% CI) of postmenopausal breast cancer in the Nurses' Health Study: individual vs joint effects of lifestyle risk factors. All PAR% estimates are based on the cumulative averaged exposure from repeated measurements

Using the comparative risk assessment method, we examined the effect of defining the low-risk group by the joint combination of the risk factors, as opposed to estimating the individual PAR% for each risk factor and then summing those PAR% to estimate the overall PAR%. When considered simultaneously, the combined PAR% of multiple risk factors tended to be higher than the product sum of the independent PAR%s (Fig. 2). This pattern was consistent whether the synergistic effect was modeled explicitly in the comparative risk assessment model or implicitly in the comparative incidence rate model (overall PAR% = 31.2% and 29.3%, respectively) versus the individual, piecemeal effect for the comparative risk assessment or Levin’s formula (18.9% and 18.6%, respectively). This trend holds true for the comparative risk assessment models of every timing, and we further observed that the difference became larger when repeated measurements were used (Table 4).

**Table 4:** Prevalence, multivariable^a^ relative risks (RR) and 95% confidence intervals (CI) for postmenopausal breast cancer, and population attributable risk (PAR%) by the comparative risk assessment method for alcohol consumption, body mass index (BMI), fruit and vegetable intake, and physical activity in the Nurses’ Health Study

		Baseline	Simple update^b^	Cumulative average^c^
**High and low risk groups were defined for each risk factor individually**				
	No. of cases	6708	6708	6708
Alcohol consumption (> 0 g/day)	High-risk%	63.6%	57.4%	72.9%
	RR (95% CI)	1.07 (1.02–1.13)	1.12 (1.06–1.18)	1.11 (1.05–1.17)
BMI (≥ 25 kg/m^2^)	High-risk%	42.6%	52.9%	52.9%
	RR (95% CI)	1.15 (1.09–1.21)	1.19 (1.13–1.25)	1.19 (1.13–1.25)
Fruit and vegetable intake (< 5 servings/day)	High-risk%	59.7%	62.2%	60.3%
	RR (95% CI)	1.04 (0.99–1.09)	1.08 (1.03–1.14)	1.05 (1.00–1.11)
Physical activity (< 18 total MET-hr/week)	High-risk%	74.0%	66.6%	66.4%
	RR (95% CI)	1.01 (0.96–1.07)	1.04 (0.98–1.09)	1.02 (0.97–1.07)
Total	Overall PAR% (95% CI)	12.5% (1.6%–23.2%)	20.7% (10.7%–30.3%)	18.9% (7.9%–29.5%)
**High and low risk groups were defined by the joint classification of the four risk factors**				
	No. of cases	6708	6708	6708
High-risk group^d^	Prevalence	97.8%	97.2%	98.1%
	RR (95% CI)	1.16 (0.98–1.38)	1.40 (1.18–1.65)	1.46 (1.19–1.80)
	PAR% (95% CI)	13.7% (-1.2%−28.1%)	28.0% (15.8%−39.3%)	31.2% (16.5%−44.5%)

*RR* relative risk

^a^Multivariable model includes: age (< 50, 50–54, 55–59, 60–64, 65–69, 70–74, 75–79, ≥ 80 years), height (< 1.60, 1.60–1.64, 1.65–1.69, 1.70–1.74, ≥ 1.75 m), age at menarche (< 12, 12, 13, 14, > 14 years), duration of oral contraceptive use (no use, > 0–2, > 2–5, > 5–10, > 10 years), joint classification of age at first birth (AFB) and parity (nulliparous, 1–2 children and AFB < 25 years, 1–2 children and AFB 25- < 30 years, 1–2 children and AFB ≥ 30 years, 3–4 children and AFB < 25 years, 3–4 children and AFB 25- < 30 years, 3–4 children and AFB 30 + years, others), menopausal status and menopausal hormone therapy use (premenopausal/unknown menopausal status, never users among postmenopausal women, past users among postmenopausal women, and current user among postmenopausal women), history of benign breast disease (yes, no), family history of breast cancer (yes, no), total energy intake (kcal/d, quintiles). Missing value for the covariates were filled in by carrying-forward responses from the last questionnaire for analyses using repeated measurements

^b^The measurement from the most recent questionnaire return for each follow-up cycle

^c^The average of all past measurements for each follow-up cycle

^d^Low-risk is defined as being at the optimal level for all four risk factors for alcohol, BMI, fruit and vegetable, and physical activity while all other participants were considered to be at high-risk

#### Sensitivity analyses

In sensitivity analysis, we corrected for potential over-reporting in fruit and vegetable intake as an example to evaluate how much it could affect the PAR% [17]. After calibration, when using Levin’s formula, the estimated PAR% in NHS went up by 4% but remained lower than that estimated for the general US population. To minimize the bias by other modifiable risk factors, we restricted the population to non-MHT users in a sensitivity analysis; the estimated PAR% remained similar (data not shown).

## Discussion

Great variability was observed in the review of 62 published PAR% estimates of lifestyle risk factors on breast cancer. The strength of the risk factor associations in the study population, the prevalence of the high-risk group, and the specific lifestyle risk factors examined contribute substantially to this variability. However, methodologic differences in how the PAR% were calculated also contributed to the inconsistency. Due to the heterogeneity of the chosen reference level, the effect size estimates, and the granularity of the prevalence data, it was not feasible to identify the specific source of variation of the PAR% estimates on the study level. Our analyses provided a series of well-controlled comparisons to formally assess the influence by each contributor on the PAR%.

### Little influence by choice of statistical method

In practice, Levin’s formula is widely used due to its simplicity but at the same time relies on approximation and strong assumptions; the comparative incidence method is intuitive and easy to adjust for age structures across populations, but would usually require having access to primary cohort data; the comparative risk assessment gives better precision of the estimation but is computationally demanding. For the overall PAR% of the risk factors in the present study, the preventable fraction did not differ much by the choice of statistical method if holding the reference level, the target population, and the timing of exposure assessment consistent. This reassured the robustness of the three methods no matter if we compared the incidence rate between the high- and low-risk populations, or if we calculated the PAR% based on the RR and the prevalence data using the Levin’s formula or the comparative risk assessment model.

### Repeated measurements demonstrated higher PAR% than a single measurement

In the literature review, only 2 NHS-based studies used repeated measurements [3, 9]. Also using NHS data, our study was able to examine different PAR% using single versus repeated measures while holding other conditions the same. The overall PAR% were generally higher by at least 50% when repeated measures were used, mainly because the repeated measurements capture the level of time-varying exposures more precisely as it reduces measurement error. Therefore, the RRs were greater in magnitude and the corresponding prevalences of the low-risk group were lower. It is our assumption that the models with more sophisticated exposure information have less error due to approximation in calculation and have reduced non-differential measurement error, and thus are able to provide PAR% estimates closer to the true attributable fraction. Moreover, repeated measures of lifestyle factors are time-integrated, suggesting preventability by long-term sustained behavioral changes.

In practice, meta-analyses and cohort studies are the two main sources of relative risk data when estimating PAR% for the general population. We estimated the overall PAR% using Levin’s formula with inputs from either meta-analyses or the NHS cohort. Similar relative risks were obtained from the meta-analyses and from the repeated measurement model from the cohort, which is in line with a recent study that showed very similar effect estimate for multiple exposures and risk of cancer in NHS compared to estimates from meta-analyses of the broad literature [34]. In summary, the pattern seen across methods suggests that PAR% could be underestimated by as much as 50% in cohort studies with single baseline measures and long follow-up, and thus any inference to the larger population should be interpreted with caution.

### Jointly, healthy lifestyle factors yield greater PAR% than the sum of individual effects

Only one study was identified that have estimated the combined PAR% of multiple factors while not assuming independence, in which the overall PAR% tended to be higher than if they had calculated the overall PAR% assuming independence [35]. Our study shows the difference in PAR% magnitude with and without the independence assumption. Methods defining the low-risk group by joint classification yielded more than 50% higher PAR% estimates than methods that considered each risk factor individually and then combined the results. The reasons could be two-fold. One could be that the combined effect of multiple risk factors may be synergistic; when additional benefit exists for maintaining multiple risk factors at the optimal levels (i.e. a significantly protective RR associated with the interaction terms), the combined PAR% is expected to be larger than the sum of the individual PAR%s. Another reason could be that the joint low-risk group has more extreme healthy behaviors due to the correlations between the risk factors. For example, in the individual method, the low-risk group for BMI would include all individuals with BMI < 25 kg/m^2^. In the jointly defined low-risk group, the individuals must have BMI < 25 kg/m^2^ and also be more active, consume less alcohol and consume more fruits and vegetables. In this study, participants in the jointly defined low-risk group on average engaged in 3 more MET-hours of activity per week, were non-drinkers (by definition), consumed 0.5 more servings of fruits and vegetables per day, and attained 50% less weight gain in adulthood compared to when each factor was evaluated individually. Although it is difficult to achieve multiple healthy behaviors, it has been shown that success in achieving one in an overall healthy lifestyle facilitated in improving others [36]. The WCRF/AICR recommendations are intended to be a package, so the overall PAR% referencing the combined low-risk level is within the spirit of what can be theoretically achieved by adhering to all recommendations.

### Limitations

There are limitations in our study. Measurement error is a concern—although systematic over-reporting would not affect the relative ranking of the participants, it did change the prevalence of each category. If, for example, fruit intake is overestimated, it would appear that more women are achieving optimal intakes than in reality, which would underestimate the potential for further risk reduction. Based on the sensitivity analyses, we have reached the conclusion that such potential impact should be limited. On the other hand, if the definition of the exposure (i.e., the cutoff point for high-risk status) changes, it would impact not only the prevalence of the population with elevated risk, but also the RR. We did not investigate the implications of this effect in all the models, but a previous study of colorectal cancer using the same cohort data have found that varying sources of RRs changed the PAR% values considerably, whereas the changes in prevalence within reasonable ranges compatible with the literature had relatively less influence on the PAR% [37]. This, again, highlights the importance to use unified definition of risk factors across studies, which has been a major endeavor of the comparative risk assessment collaborating group [10]. Lastly, the study population consists of predominately White female nurses, which may limit the validity of extrapolation to other populations. To complete the analysis, we supplemented the study with prevalence data from national surveys, and the results indeed suggest that the prevalence differences between NHS and NHANES/NHIS would cause large difference in PAR% estimations, which reveals another important source of variation. Nevertheless, the main purpose of this study was to leverage the strong cohort data to demonstrate the sources and degree of variation in the computation of PAR% rather than optimizing its generalizability.

## Conclusions

Our results suggest that the three statistical methods, given the same data source, provide similar results. However, sizable increases in the PAR% were observed when repeated measures were used instead of a single measure, as well as for calculations based on women achieving all recommendations rather than considering each individually and then combining the results. Thus, PAR%s in the current literature have likely underestimated the preventable fraction of postmenopausal breast cancer. These results emphasize the importance of high-quality data sources, call for cautious interpretation of PAR% in the current literature as the risk factors we examined likely has a larger impact in preventing lifestyle-related diseases, and underscore the potential benefit on disease risk of long-term behavioral change toward an overall healthier lifestyle.

## Supplementary Information

Below is the link to the electronic supplementary material.

Supplementary file1 (DOCX 518 kb)
