# Supplementary material for: Influence of analytic methods, data sources, and repeated measurements on the population attributable fraction of lifestyle risk factors
Source: Eur J Epidemiol. 2023 Jun 6;38(7):717–28. doi: 10.1007/s10654-023-01018-z (PMC10275810; doi:10.1007/s10654-023-01018-z)
Supplement: Supplementary file 1 — Supplementary file1 (DOCX 518 kb) [file 10654_2023_1018_MOESM1_ESM.docx]

**Table S1. Databases and search strategy for systematic literature review**

| Database | Search strategy |
| --- | --- |
| Pubmed | 1900/01/01:2022/09/30[Date - Publication] AND "breast neoplasms"[MeSH Major Topic] AND ((("populate"[All Fields] OR "populated"[All Fields] OR "populates"[All Fields] OR "populating"[All Fields] OR "population"[MeSH Terms] OR "population"[All Fields] OR "population groups"[MeSH Terms] OR ("population"[All Fields] AND "groups"[All Fields]) OR "population groups"[All Fields] OR "populations"[All Fields] OR "population s"[All Fields] OR "populational"[All Fields] OR "populous"[All Fields]) AND "attributable*"[All Fields]) OR "case avert*"[All Fields] OR "preventable fraction"[All Fields]) AND ("life style"[MeSH Major Topic] OR "diet"[Title/Abstract] OR "nutrition"[Title/Abstract] OR "physical activity"[Title/Abstract] OR "obesity"[MeSH Major Topic] OR "BMI"[Title/Abstract]) |
| Embase | ('population attributable fraction' OR 'population attributable risk') AND 'breast cancer' AND ('lifestyle' OR 'diet' OR 'obesity' OR 'nutrition' OR 'physical activity') |
| Web of Science | DOP=(1900-01-01/2022-09-30) AND (TS=("PAF") OR TS=("PAR")) AND TS=(breast cancer) AND ((TS=lifestyle) OR TS=(obesity) OR TS=(BMI) OR TS=(diet) OR TS=(physical activity) OR TS=(nutrition)) |


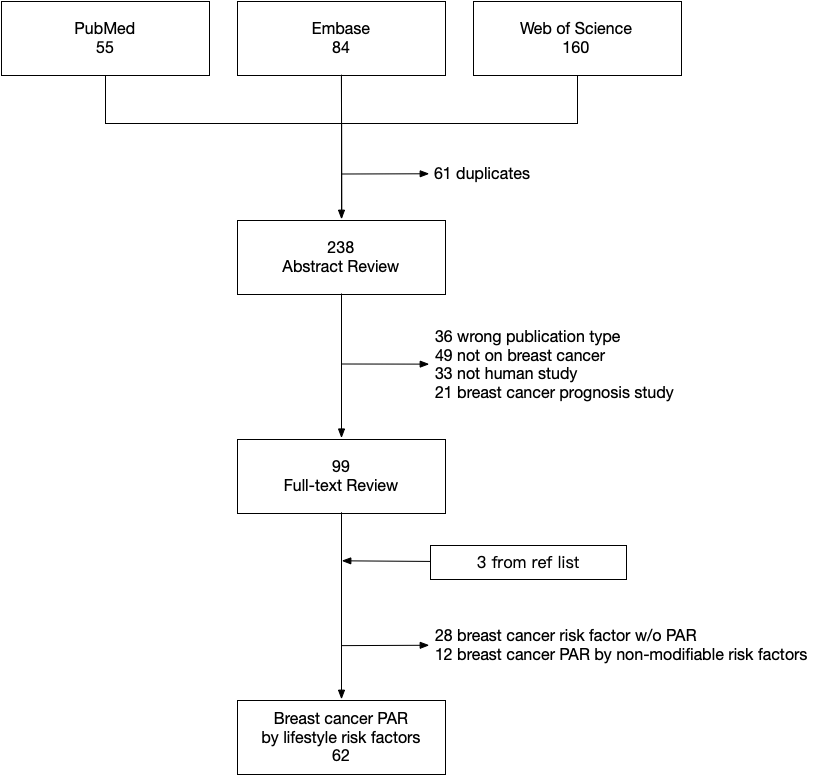


**Figure S1. Flow chart of literature screening**

**Figure S2. Nurses’ Health Study cohort data collection and the modeling of exposures.**

Diet: alcohol, fruit and vegetable intake; wt: weight; PA: physical activity. The solid circles represent exposure measurements; the arrows refer to follow-up periods.

**
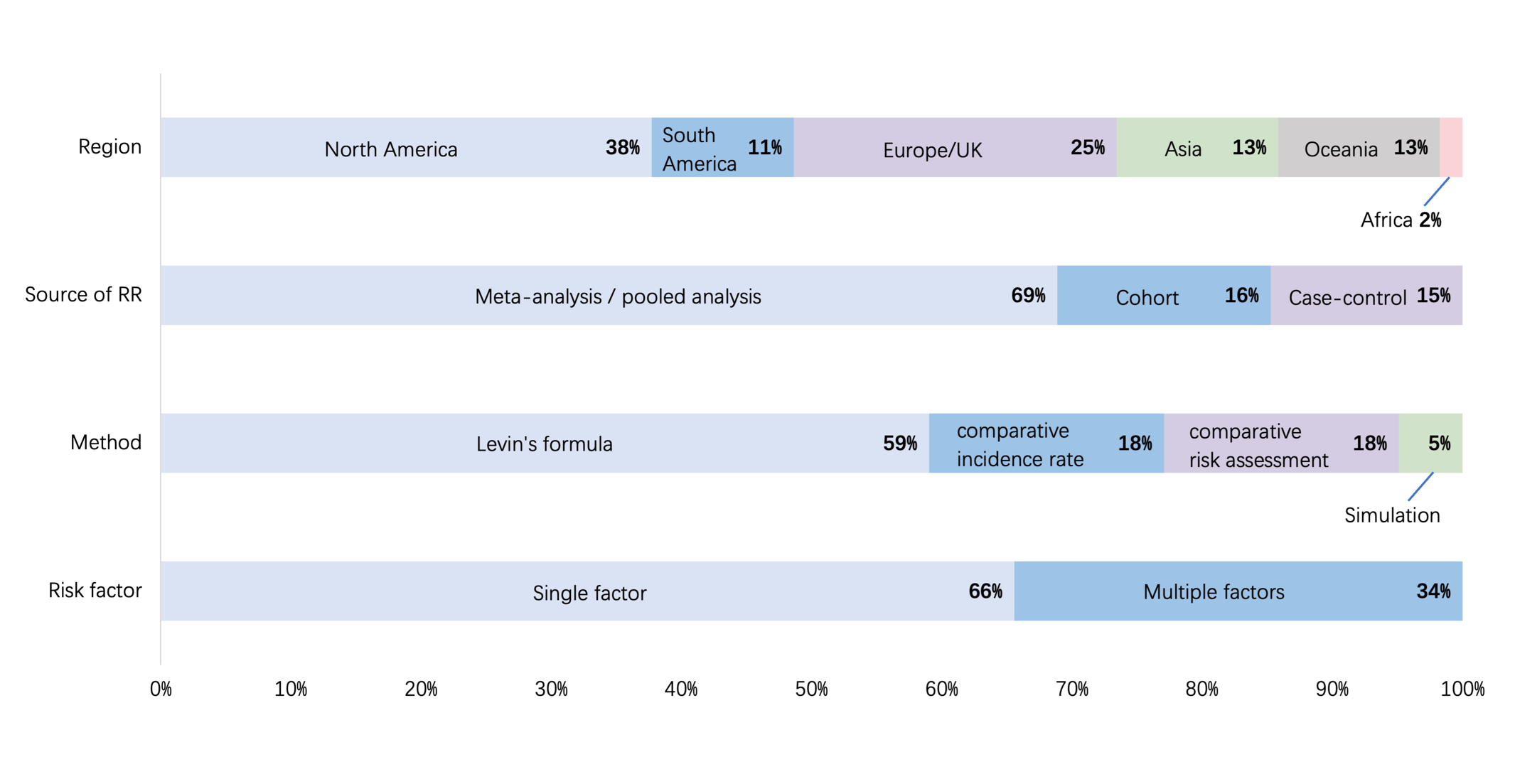
**

**Figure S3. Summary of studies on PAR% of breast cancer by modifiable lifestyle risk factors**

**Table S2. Characteristics of studies on PAR% of breast cancer by lifestyle risk factors**

| Author | Year | Region | Source of prevalence | Source of RR | Measurement | Follow-up (yr) | Method | Factors | Reference level | PAR% |
| --- | --- | --- | --- | --- | --- | --- | --- | --- | --- | --- |
| Sprague(37) | 2008 | US | Cancer registry-based case-control study: Collaborative Breast Cancer Study | Case-control (OR) | Single | - | Comparative incidence rate | Alcohol, Weight gain, PA | Alcohol: 1 drink/d Weight gain since 18: 5kg PA: 5h/wk | Alcohol: 6.1% Weight gain: 21.3% PA: 15.7% |
| Engmann(38) | 2017 | US | Cancer registry-based case-control study: Breast Cancer Surveillance Consortium (BCSC) | Case-control (OR) | Single | - | Comparative incidence rate | BMI | BMI: 25 kg/m^2^ | BMI: 16.4% |
| Ghiasvand(39) | 2012 | Iran | Hospital-based case–control study | Case-control (OR) | Single | - | Comparative incidence rate | BMI | BMI: 25 kg/m^2^ | BMI: 24.8% |
| Ho(40) | 2020 | Singapore | Population-based prospective trial: The Singapore Breast Cancer Screening Project (SBCSP) | Case-control (OR) | Single | - | Comparative incidence rate | BMI | BMI: 25 kg/m^2^ | BMI: 17.7% |
| La Vecchia(41) | 1997 | Italy | Cooperative pooling of 3 Italian case-control studies | Case-control (OR) | Single | - | Comparative incidence rate | BMI | BMI: 25 kg/m^2^ | BMI: 19.6% |
| Barnes(42) | 2011 | Germany | Population-based case-control study: the Mammary carcinoma Risk factor Investigation (MARIE) | Case-control (OR) | Single | - | Comparative incidence rate | HT, PA, BMI Alcohol | HT: past user PA: 76.5 MET-hr/wk BMI: 22.5 kg/m^2^ Alcohol: 0 | HT, 19.4% PA, 12.8% BMI, 2.4% Alcohol, -7.6% |
| Mezzetti(35) | 1998 | Italy | Multicenter case-control study of breast cancer in six areas in Italy | Case-control (OR) | Single | - | Comparative incidence rate | β-Carotene, Vitamin E, Alcohol, Physical activity, BMI | β-Carotene: 3366 μg/d Vit E: 8.5 mg/d Alcohol: 20 PA: low BMI: 23.3 kg/m^2^ | β-Carotene: 11.5% Vitamin E: 8.3% Alcohol: 5.4% Physical activity: 14.4% BMI: 10.2% |
| Park(43) | 2016 | Korea | Nationally representative case-control study: the Korea National Health and Nutrition Examination Surveys (KNHANES) | Case-control (OR) | Single | - | Levin's formula | BMI, PA, Alcohol | BMI: 25 kg/m^2^ PA: yes Alcohol: 0 | BMI: 1% PA: 28.8% Alcohol: 5.1% |
| Castelló(44) | 2017 | Spain | Case-control study of 10 Spanish provinces: MCC-Spain | Case-control (OR) | Single |  | Levin's formula | Western diet, Mediterranean diet | Western diet: Q1; Med diet: Q4 | Western diet: 20% Mediterranean diet: 18% |
| Song(33) | 2016 | US | Prospective cohort study: The Nurses’ Health Study (NHS) | Cohort | Biennially | 24-30 | Comparative incidence rate | Smoking, PA, BMI, Alcohol | Smoking: no or past;  Alcohol: ≤1 drink/d for women, ≤2 drinks/d for men;  BMI: 27.5 kg/m^2^;  PA: 7.5 metabolic-equivalent [METs] hours per week | NR |
| da Silva(45) | 2021 | Norway | Nationally representative prospective cohort: The Norwegian Women and Cancer (NOWAC) study | Cohort | Twice to calculate 7-yr weight change | 16.2 | Comparative Risk Assessment | Weight gain | Weight gain: 2kg | Weight gain: 16.8% |
| Bissell(46) | 2020 | US | U.S.-based Breast Cancer Surveillance Consortium (BCSC) of 7 registries | Cohort | Baseline | 5.2 | Comparative Risk Assessment - Spiegelman | BMI | BMI: 25 kg/m^2^ | White: 15.4% Black: 28.3% Asian: 14.7% Hispanic: 12.0% |
| Masala(11) | 2017 | Italy | Five Italian cohorts of the European Prospective Investigation into Cancer and Nutrition (EPIC) prospective study | Cohort | Baseline | 14.8 | Comparative Risk Assessment - Spiegelman | PA, Alcohol, BMI | PA: 151.0 MET hours/week;  Alcohol: 10 g/day;  BMI: 25 kg/m^2^ | PA: 14% Alcohol: 7% BMI: 12% |
| Tamimi(9) | 2016 | US | Prospective cohort study: The Nurses’ Health Study (NHS) | Cohort | Biennially | 30 | Comparative Risk Assessment - Spiegelman | Weight gain, Alcohol, PA | Weight gain: 2kg Alcohol: 0 PA: highest quartile | Weight gain: 18.7% Alcohol: 5.8% PA: 3.3% |
| Lee(47) | 2019 | Korea | Nationwide cohort: the National Health Insurance Service (NHIS) claims | Cohort | Baseline | 13 | Levin's formula | BMI | BMI: 23 kg/m^2^ | BMI: 18.6% |
| Gram(48) | 2016 | Norway | Nationally representative prospective cohort: The Norwegian Women and Cancer (NOWAC) study | Cohort | Baseline | 13.4 | Levin's formula | Smoking | Smoking: no | Passive smoking: 3.2% Active smoking: 11.9% |
| Al-Ajmi(49) | 2018 | UK | National-based cohort: UK Biobank | Cohort | Baseline | 9.8 | STATA punaf | BMI | BMI: 25 kg/m^2^ | BMI: 8.3% |
| Arriaga(50) | 2019 | Australia | National population-based cohorts: AusDiab Study (1999–2000), the LADY (Learning how Australians Deal with menopause sYmptoms) Survey (2013), and the Australian National Health Surveys (NHS 2014–2015 and 2001) | Cohort:  Australian cancer-PAF cohort consortium of 7 cohort studies | Baseline | 4.7 | Comparative Risk Assessment | Alcohol, BMI | Alcohol: 1 drink/d BMI: 25 kg/m^2^ | Alcohol, 6.6%  BMI: 12.8% |
| Petrelli(51) | 2002 | US | National representative survey: The third National Health and Nutrition Examination Survey (NHANES III) | Cohort | Baseline | 14 | Levin's formula | BMI | BMI: 20.5 kg/m^2^ | BMI: 49.3% |
| Tseng(52) | 1999 | US | Nationally representative survey: NHANES III | meta-analysis | - | - | Comparative incidence rate | Alcohol | Alcohol: 0 | Alcohol: 2.1% (NHANES) or 7.4% (NHS) |
| Wienecke(53) | 2015 | Germany | Nationwide sample: the German Health Interview and Examination Survey for Adults (DEGS1) | meta-analysis | - | - | Comparative incidence rate | Alcohol | Alcohol: 0 | Alcohol: 6.6% |
| Rezende(54) | 2021 | Brazil | National representative health survey: the National Health Survey (Pesquisa Nacional de Saúde – PNS 2013) | meta-analysis | - | - | Comparative Risk Assessment | PA | PA: ideal = ≥8000 MET-min/week guideline achievable = ≥600 MET-min/week | PA ideal: 11.8% PA guideline: 1.7% |
| Rezende(55) | 2018 | Brazil | National representative health survey: the National Health Survey (Pesquisa Nacional de Saúde – PNS 2013) | meta-analysis | - | - | Comparative Risk Assessment | PA | PA, 5 scenarios:  ideal = ≥8000 MET-min/week, guideline achievable = ≥600 MET-min/week, 10% improvement, All level up to most active state, Females level up to males | PA, 5 scenarios:  ideal = ≥8000 MET-min/week: 12.29%, guideline achievable = ≥600 MET-min/week: 1.74%, 10% improvement: 0.17%, All level up to most active state: 0.33%, Females level up to males: 1.09% |
| Poirier(56) | 2019 | Canada | National representative survey: Canadian Community Health Survey (CCHS) | meta-analysis | - | - | Levin's formula | Active/passive tobacco smoking, Physical inactivity, Excess weight, Sedentary behaviour, Alcohol. | Active tobacco smoking: no Passive tobacco smoke: no  Physical inactivity: no Excess weight, Sedentary behaviour, Alcohol consumption: 0 | Active tobacco smoking: 4.6% Passive tobacco smoke: 1.3% Physical inactivity: 7.4% Excess weight:4.1% Sedentary behaviour: 2.8% Alcohol: 3.2% |
| Kehoe(57) | 2012 | Global | International alcohol consumption survey: Gender, Alcohol and Culture: An International Study (GENACIS), from the European Comparative Alcohol Study (ECAS), and from the STEPwise approach to Surveillance (STEPS) | meta-analysis | - | - | Levin's formula | Alcohol | Alcohol: 0 | Alcohol: 2.3% in Israel - 20.1% in Brazil |
| Park(58) | 2014 | Korea | National representative survey: Korean National Health Examination Surveys (KNHES) | meta-analysis | - | - | Levin's formula | Alcohol | Alcohol: 0 | Alcohol: 0.2% |
| Hayes(8) | 2013 | New Zealand | Population-based surveys: the 2006/07 New Zealand Health Survey and the 2008/09 New Zealand Adult Nutrition Survey. | meta-analysis | - | - | Levin's formula | Alcohol, BMI, Diet, PA, Smoking | Alcohol: 0 BMI:25 kg/m^2^ PA: active (≥30min/wk) | Alcohol:  Maori 7.2%;  Pacific 4.2%;  European 3.1%. BMI:  Maori 15.9%;  Pacific 16.9% ;  European 9.5%. PA:  Maori 3.1%; Pacific 4.4%; European 3.2%. |
| Whiteman(59) | 2015 | Australia | 2001 National Health Survey Confidentialised Unit Record Files | meta-analysis | - | - | Levin's formula | Alcohol, BMI, PA | Alcohol: 0 BMI: 25 kg/m^2^ PA: 5 - 30 MET-hr/wk of moderate activity | Alcohol: 5.9% BMI: 6.8% PA: 6.8% |
| Wilson(60) | 2018 | Australia | Population-based survey: the National Health and Nutrition Surveys conducted by the Australian Bureau of Statistics | meta-analysis | - | - | Levin's formula | Alcohol, BMI, PA | Alcohol: 0 BMI: 25 kg/m^2^ PA: 5 - 30 MET-hr/wk of moderate activity | Alcohol: 4.8% BMI: 7.3% PA: 7.2% |
| Wilson(61) | 2013 | Queensland, Australia | Cross-sectional prevalence survey: Queensland Screening and Assessment Services (BreastScreen Queensland survey) | meta-analysis | - | - | Levin's formula | Alcohol, BMI, PA | Alcohol: 0 BMI: 25 kg/m^2^ PA: 27 MET-hr/wk | Alcohol: 2.8% BMI: 12.1% PA: 7.6% |
| Brenner(62) | 2017 | Alberta - Canada | National representative survey: Canadian Community Health Survey (CCHS) | meta-analysis | - | - | Levin's formula | BMI | BMI: 25 kg/m^2^ | BMI: 8.0% |
| Collins(63) | 2017 | Ireland | National population survey: the Survey of Lifestyles Attitudes and Nutrition (SLAN) 2002 | meta-analysis | - | - | Levin's formula | BMI | BMI: 25 kg/m^2^ | BMI: 8% |
| Islami(64) | 2019 | US | Nationwide telephone survey of adults 18 years or older: the 2001 to 2004 Behavioral Risk Factor Surveillance System (BRFSS), 2001 to 2004 data from the National Health and Nutrition Examination Survey (NHANES) | meta-analysis | - | - | Levin's formula | BMI | BMI: 25 kg/m^2^ | NR |
| Luisa Garmendia(65) | 2013 | Chile | National representative survey: La Encuesta Nacional de Salud del año 2010 | meta-analysis | - | - | Levin's formula | BMI | BMI: 25 kg/m^2^ | BMI： 17% |
| Luo(66) | 2007 | Canada | Six national population- based health surveys: the 1970-1972 Nutrition Canada Survey, the 1978-1979 Canada Health Survey, the 1981 Canada Fitness Survey, the 1988 Campbell’s Survey on Well-being in Canada, the 1986-1992 Canadian Heart Health Surveys and the 2004 Canadian Community Health Survey (CCHS), Cycle 2.2. | meta-analysis | - | - | Levin's formula | BMI | BMI: 30 kg/m^2^ | BMI: 12.09% |
| Brenner(67) | 2014 | Canada | National representative survey: Canadian Community Health Survey (CCHS) | meta-analysis | - | - | Levin's formula | BMI, PA | BMI: 25 kg/m^2^ PA: 3 kcal/kg/d | BMI:7.5% PA: 18.5% |
| Neutel(68) | 2010 | Canada | National representative survey: The National Population Health Survey (NPHS) | meta-analysis | - | - | Levin's formula | BMI, Alcohol, Smoking, PA | BMI: 30 kg/m^2^,  Alcohol: 9 drinks/wk Smoking: yes PA: active | In 2006, BMI: 8.8% Alcohol: 2.6% Smoking: 3.1% PA: 6.4% |
| Park(69) | 2014 | Korea | Large-scale population-based cohort study: the National Health Insurance Corporation 1992-1995, and 1989 Korea National Health Examination Survey (KNHES) | meta-analysis | - | - | Levin's formula | BMI, PA | BMI: 23 kg/m^2^ PA: light-moderate | BMI: 8.2% PA: 8.8% |
| van Gemert(70) | 2015 | Netherland | National surveys: The intake of energy and nutrients of food groups by the Dutch population. Food consumption survey 1997–1998.  Statistics Netherlands (CBS) (2001) Permanent national survey on the situation of life (POLS). Mulier Institute the Netherlands (2000) Survey on accidents and physical activity in the Netherlands (OBIN).  STIVORO. TNS NIPO (2014) Smoking behavior in adults. | meta-analysis | - | - | Levin's formula | BMI, PA, Alcohol, Smoking, Fiber | BMI: 25 kg/m^2^ PA: active Alcohol: 0 Smoking: no Fiber: 27g/d | BMI: 8.8% PA: 5.5% Alcohol: 6.6% Smoking: 4.6% Fiber: 3.2% |
| Katzmarzyk(71) | 2022 | Global | Pooled analysis of 358 population-based surveys with 1.9 million participants globally | meta-analysis | - | - | Levin's formula | PA | PA: 7.5 to 14.9 MET-h/wk | PA: 2.8% |
| Katzmarzyk(72) | 2000 | Canada | Nationally representative survey: Physical Activity Monitor Survey | meta-analysis | - | - | Levin's formula | PA | PA: active | PA: 11.0% |
| Machado de Rezende(73) | 2015 | Brazil | National Household Survey: 2008 Brazilian Pesquisa Nacional por Amostra de Domicí- lios (PNAD) | meta-analysis | - | - | Levin's formula | PA | PA: any activity | PA: 5.18% |
| Katzmarzyk(74) | 2004 | Canada | 2000/2001 Canadian Community Health Survey. | meta-analysis | - | - | Levin's formula | PA, BMI | PA: active BMI: 22.5-25 kg/m^2^ | PA: 14.2% BMI: 6.5% |
| Chang(75) | 2012 | US | National Health and Nutrition Examination Survey (NHANES) | meta-analysis | - | - | Simulation model - CISNET | BMI | BMI: 30 kg/m^2^ | White: 4.5% or 3.9% Black: 3.6% or 2.5% (SPECTRUM vs MISCAN model) |
| Behrens(76) | 2018 | Germany | Nationally representative German Health Interview and Examination Survey for Adults for the period 2008 to 2011 (DEGS1) | meta-analysis | - | - | Levin's formula | BMI, PA, Diet | BMI: 25 kg/m^2^ PA: 150 min/wk Fiber: 32g/d Processed meat: 0 | BMI: 9.0% PA: 7.1% Fiber: 8.8% Processed meat: 5.2% |
| Clarke(77) | 2006 | California, US | Population-based survey - the California Health Interview Survey (CHIS) | pooled analysis | - | - | Levin's formula | Alcohol, PA | Alcohol: 2 beverages PA: no vigorous/moderate activity | Alcohol: 3.5% PA: 7.5% |
| Minihan(78) | 2022 | US | Nationwide telephone survey of adults: The Behavioral Risk Factor Surveillance System (BRFSS) | pooled analysis | - | - | Levin's formula | PA | PA: ≥15 MET·h/wk | PA: 6.5% |
| Arnold(79) | 2015 | Global | Global Burden of Metabolic Risk Factors of Chronic Diseases Collaborating Group (GBMRF) | WCRF CUP meta-analysis | - | - | Comparative Risk Assessment | BMI | BMI: 25 kg/m^2^ | Sub-Saharan Africa 25%  Middle East and Northern Africa 48%  Latin America & Caribbean 42% North America 48% East Asia 20% South-East Asia 19%  South-Central Asia 17% Eastern Europe 45%  Northern Europe 42% Southern Europe 41%  Western Europe 41%  Oceania 42% |
| Arnold(80) | 2018 | France | Nationally representative cross-sectional dietary and health survey: 2006 National Nutrition and Health Survey | WCRF CUP meta-analysis | - | - | Comparative Risk Assessment | BMI | BMI: 22 kg/m^2^ | BMI: 10.6% |
| de Carvalho(81) | 2022 | Brazil | National survey: Brazilian National Health Survey (NHS) | WCRF CUP meta-analysis | - | - | Comparative Risk Assessment | BMI | BMI: 22 kg/m^2^ | BMI: 8% |
| Goodarzi(82) | 2019 | Asia | NR | WCRF CUP meta-analysis | - | - | Comparative Risk Assessment | BMI | BMI: 22 kg/m^2^ | see tables for 33 countries |
| Mons(83) | 2018 | Germany | National survey: German Health Interview and Examination Survey for Adults for the period 2008 to 2011 (DEGS1 2008–2011) | WCRF CUP meta-analysis | - | - | Levin's formula | Alcohol | Alcohol: 10g/d | Alcohol: 1.3% |
| da Silva(84) | 2021 | Brazil | National survey: 2008–2009 National Household Budget Survey | WCRF CUP meta-analysis | - | - | Levin's formula | BMI | BMI: 25 kg/m^2^ | BMI: 5.16% |
| Kendall(85) | 2015 | Australia | National survey: 2001 National Health Survey, Australia | WCRF CUP meta-analysis | - | - | Levin's formula | BMI | BMI: 25 kg/m^2^ | BMI: 7.8% |
| Zakaria(86) | 2017 | Canada | Population-based survey: the Canadian Community Health Survey (CCHS) | WCRF CUP meta-analysis | - | - | Levin's formula | BMI | BMI: 25 kg/m^2^ | BMI: 9.7% |
| Poirier(87) | 2019 | Canada | Population-based survey: the 2003 Canadian Community Health Survey (CCHS) | WCRF CUP meta-analysis | - | - | Levin's formula | Fruits & Vegetables | Fruits & Vegetables: 4+ servings/day | Fruits & Vegetables: 5.8% |
| Olsen(88) | 2015 | Australia | National survey: 2001 National Health Survey Confidentialised Unit Record Files | WCRF CUP meta-analysis | - | - | Levin's formula | PA | PA: 30 MET-hours/wk | PA: 6.8% |
| Parkin(89) | 2011 | UK | National survey: The National Diet and Nutrition Survey (FSA, 2004) | WCRF CUP meta-analysis | - | - | Levin's formula | PA | PA: 30 min, 5 days a week, | PA: 3.4% |
| Touillaud(90) | 2019 | France | National cross-sectional survey: the National Nutrition and Health Survey (Etude nationale nutrition santé, ENNS) | WCRF CUP meta-analysis | - | - | Levin's formula | PA | PA: 21 MET-hours per week | PA: 3.8% |
| Andersson(91) | 2017 | Nordic countries | National Registry Based Survey: The Nordic monitoring system on diet, PA, and overweight | WCRF CUP meta-analysis | - | - | The Prevent macrosimulation model | BMI | BMI: 25 kg/m^2^ | BMI: 7% |
| Andersson(92) | 2019 | Nordic countries | National Registry Based Survey: The Nordic monitoring system on diet, PA, and overweight. | WCRF CUP meta-analysis | - | - | The Prevent macrosimulation model | PA | PA: 15 MET-hr/wk | PA: 0.6% |

Abbreviations: BMI: body mass index; PA: physical activity; MET: metabolic equivalent of task; hr: hour; d: day; wk: week; NR: not reported.

| **Table S3. Distribution of lifestyle risk factors for postmenopausal breast cancer in the Nurses' Health Study (n=100,148)** | | | | | | | | |
| --- | --- | --- | --- | --- | --- | --- | --- | --- |
| Risk factors | | Frequency (%) | |  | Covariates | | Frequency (%) | |
| Modifiable risk factors in WCRF recommendations [6] | | | |  | Non-modifiable risk factors and covariates | | |  |
|  |  | Baseline (1986) | Cumulative average |  |  |  | Baseline (1986) | End-of-follow-up |
| Alcohol consumption | |  |  |  | Parity and age at first birth (AFB) | |  |  |
|  | nondrinker | 35.1% | 27.1% |  |  | Nulliparous | 5.8% | 5.7% |
|  | >0-<5 g/d | 32.8% | 41.5% |  |  | 1-2 children AFB <25yr | 14.0% | 13.8% |
|  | 5-<10 g/d | 11.0% | 12.7% |  |  | 1-2 children AFB 25-<30yr | 15.4% | 15.0% |
|  | 10-<15 g/d | 9.4% | 7.9% |  |  | 1-2 children AFB ≥30yr | 6.3% | 6.2% |
|  | 15-<30 g/d | 6.9% | 7.7% |  |  | 3-4 children AFB <25yr | 25.7% | 25.8% |
|  | ≥30 g/d | 4.8% | 3.3% |  |  | 3-4 children AFB 25-<30yr | 16.4% | 16.5% |
| Body mass index | |  |  |  |  | 3-4 children AFB ≥30yr | 2.6% | 2.7% |
|  | <18.5 kg/m^2^ | 1.4% | 2.1% |  |  | Others | 14.0% | 14.4% |
|  | 18.5-<25 kg/m^2^ | 56.0% | 44.9% |  | Oral contraceptive (OC) use | |  |  |
|  | 25-<30 kg/m^2^ | 28.5% | 32.6% |  |  | No OC use | 53.1% | 53.7% |
|  | ≥30 kg/m^2^ | 14.1% | 20.3% |  |  | >0-2 yrs of OC use | 17.9% | 17.6% |
| Fruit and vegetable intake | |  |  |  |  | >2-5yrs of OC use | 12.9% | 12.7% |
|  | 0-<2 servings/d | 8.5% | 5.8% |  |  | >5-10 yrs of OC use | 11.2% | 11.1% |
|  | 2-<4 servings/d | 33.7% | 34.4% |  |  | >10 yrs of OC use | 5.0% | 4.9% |
|  | 4-<6 servings/d | 31.2% | 35.7% |  | Benign breast disease history | | 29.8% | 39.2% |
|  | 6-<8 servings/d | 16.2% | 16.3% |  | Family history of breast cancer | | 8.1% | 17.8% |
|  | ≥8 servings/d | 10.4% | 7.8% |  | Menopausal status and menopausal hormone therapy use | | |  |
| Physical activity | |  |  |  |  | Premenopausal/missing menopause | 33.3% | 2.2% |
|  | <3 total MET-hrs/wk | 27.4% | 12.6% |  |  | Postmenopausal, never user | 35.4% | 39.9% |
|  | 3-<9 total MET-hrs/wk | 27.0% | 26.1% |  |  | Postmenopausal, past user | 14.3% | 43.5% |
|  | 9-<18 total MET-hrs/wk | 19.6% | 27.7% |  |  | Postmenopausal, current user | 17.1% | 14.4% |
|  | 18-<27 total MET-hrs/wk | 10.8% | 15.5% |  | Smoking status | |  |  |
|  | ≥27 total MET-hrs/wk | 15.2% | 18.1% |  |  | Never smoker | 44.1% | 44.0% |
|  |  |  |  |  |  | Past smoker | 34.8% | 47.8% |
|  |  |  |  |  |  | Current smoker | 21.1% | 8.2% |

| **Table S4. Multivariable^a^ relative risks (RR) and 95% CI of risk factors^b^ of postmenopausal breast cancer in the Nurses' Health Study** | | | | | | | | | | | |
| --- | --- | --- | --- | --- | --- | --- | --- | --- | --- | --- | --- |
| **Exposure** | **Exposure category^c^** | **Baseline** | | | **Simple update** | | | **Cumulative average** | | | |
|  |  | Prevalence (%) | RR | 95% CI | Prevalence (%) | RR | 95% CI | Prevalence (%) | RR | 95% CI |  |
| Alcohol consumption  (drink/day) | **None** | 35.1% | 1.00 | - | 42.5% | 1.00 | - | 27.2% | 1.00 | - |  |
|  | <1 | 51.2% | 1.06 | (1.00-1.12) | 44.5% | 1.09 | (1.03-1.15) | 60.5% | 1.08 | (1.02-1.15) |  |
|  | 1-<2 | 8.2% | 1.15 | (1.05-1.26) | 8.2% | 1.20 | (1.10-1.31) | 8.4% | 1.26 | (1.15-1.39) |  |
|  | ≥2 | 5.5% | 1.36 | (1.23-1.50) | 4.8% | 1.33 | (1.20-1.48) | 3.9% | 1.35 | (1.20-1.53) |  |
| BMI  (kg/m^2^) | **Normal (18.5-<25)** | 57.4% | 1.00 | - | 47.1% | 1.00 | - | 53.4% | 1.00 | - |  |
|  | Overweight (25-<30) | 28.5% | 1.15 | (1.09-1.22) | 32.6% | 1.18 | (1.11-1.24) | 31.7% | 1.18 | (1.12-1.24) |  |
|  | Obese (≥30) | 14.1% | 1.16 | (1.08-1.25) | 20.3% | 1.27 | (1.19-1.35) | 15.0% | 1.21 | (1.12-1.30) |  |
| Fruit and vegetable intake (g/day) | <200 | 8.5% | 1.07 | (0.97-1.18) | 9.9% | 1.13 | (1.04-1.24) | 5.8% | 1.17 | (1.05-1.31) |  |
|  | 200-<400 | 51.3% | 1.02 | (0.97-1.08) | 52.3% | 1.08 | (1.02-1.14) | 54.5% | 1.04 | (0.99-1.10) |  |
|  | **≥400** | 40.3% | 1.00 | - | 37.8% | 1.00 | - | 39.7% | 1.00 | - |  |
| Physical activity  (MET-hrs/week) | <4.2 | 34.1% | 1.01 | (0.95-1.08) | 27.9% | 0.96 | (0.90-1.03) | 17.3% | 0.98 | (0.91-1.06) |  |
|  | 4.2-<12.5 | 30.2% | 1.02 | (0.96-1.09) | 27.3% | 1.07 | (1.00-1.13) | 33.9% | 1.04 | (0.98-1.10) |  |
|  | 12.5-<16.7 | 9.6% | 0.97 | (0.89-1.07) | 11.4% | 1.03 | (0.95-1.12) | 15.2% | 0.98 | (0.91-1.05) |  |
|  | **≥16.7** | 26.0% | 1.00 | - | 33.4% | 1.00 | - | 33.6% | 1.00 | - |  |
| ^a^ Multivariable model includes: age (<50, 50-54, 55-59, 60-64, 65-69, 70-74, 75-79, ≥80 years), height (<1.60, 1.60-1.64, 1.65-1.69, 1.70-1.74, ≥1.75m), age at menarche (<12, 12, 13, 14, >14 years), duration of oral contraceptive use (no use, >0-2, >2-5, >5-10, >10 years), joint classification of age at first birth (AFB) and parity (nulliparous, 1-2 children and AFB <25 years, 1-2 children and AFB 25-<30 years, 1-2 children and AFB ≥30 years, 3-4 children and AFB <25 years, 3-4 children and AFB 25-<30 years, 3-4 children and AFB 30+ years, others), menopausal status and menopausal hormone therapy use (premenopausal/unknown menopausal status, never users among postmenopausal women, past users among postmenopausal women, and current user among postmenopausal women), history of benign breast disease (yes, no), family history of breast cancer (yes, no), total energy intake (kcal/d, quintiles). Missing value for the covariates were filled in by carrying-forward responses from the last questionnaire for analyses using repeated measurements.  ^b^ Risk factors with repeated measures in the Nurses' Health Study selected based on summary of strong evidence on diet, nutrition, physical activity, and the prevention of cancer 2018 by WCRF/AICR. | | | | | | | | | | | |
| ^c^ Theoretical minimum risk (reference level) in bold. | | | | | | | | | | | |

| **Table S5. Summary relative risks (RR) and 95% confidence intervals (CI) of risk factors of postmenopausal breast cancer from meta-analyses of cohort studies** | | | | | | | |
| --- | --- | --- | --- | --- | --- | --- | --- |
| Risk factor | Summary RR (95% CI) from the published literature | Summary RR (95% CI)  used in this study | Exposure category | Prevalence (%) | Summary RR | 95% CI |  |
| Alcohol consumption (30) | Per 10g/d increase | Per 1 drink/d increase | **None** | 40.7 | 1.00 | - |  |
|  | 1.10 (1.08-1.13) | 1.14 (1.11-1.19) | < 1 drink/d | 37.7 | 1.07 | (1.05-1.09) |  |
|  | or per 1 drink/d increase |  | 1-<2 drinks/d | 8.1 | 1.22 | (1.17-1.30) |  |
|  | 1.14 (1.11-1.19) |  | ≥2 drinks/d | 13.6 | 1.39 | (1.30-1.54) |  |
| BMI (31) | Per 5kg/m^2^ increase | Per 5 kg/m^2^ increase | **18.5-<25 kg/m^2^** | 28.7 | 1.00 | - |  |
|  | 1.12 (1.10-1.15) | 1.12 (1.10-1.15) | 25-<30 kg/m^2^ | 29.6 | 1.12 | (1.10-1.15) |  |
|  |  |  | ≥30 kg/m^2^ | 40.1 | 1.25 | (1.21-1.32) |  |
| Fruit and vegetable intake (32) | Per 200g/d increase | Per 200g/d decrease | <200 g/d | 49.2 | 1.08 | (1.00-1.17) |  |
|  | 0.96 (0.93-1.00) | 1.04 (1.00-1.08) | 200-<400 g/d | 41.3 | 1.04 | (1.00-1.08) |  |
|  |  |  | **≥400 g/d** | 9.4 | 1.00 | - |  |
| Physical activity (31) | Per 10 MET-hrs/wk or | per 10 MET-hrs/wk decrease | 0-249 MET-hrs/wk | 57.9 | 1.03 | (1.01-1.04) |  |
|  | per 600 MET-min/wk increase | 1.02 (1.01-1.03) | 250-749 MET-hrs/wk | 15.6 | 1.02 | (1.01-1.02) |  |
|  | 0.98 (0.97-0.99) |  | 750-999 MET-hrs/wk | 5.4 | 1.00 | (1.00-1.01) |  |
|  |  |  | **≥ 1000 MET-hrs/wk** | 21.1 | 1.00 | - |  |
|  |  |  |  |  |  |  |  |

| **Table S6. Distribution^a^ of the risk factors^b^ for postmenopausal breast cancer in the Nurses' Health Study (NHS), the National Health and Nutrition Examination Survey (NHANES), and the National Health Interview Survey (NHIS)** | | | | | | |
| --- | --- | --- | --- | --- | --- | --- |
|  | NHS | | | | NHANES & NHIS^c^ | |
|  | Category | Prevalence (%) | | | Category^f^ | Prevalence (%) |
|  |  | Baseline | Simple update^d^ | Cumulative average^e^ |  |  |
| Alcohol consumption |  |  |  |  |  |  |
|  | **0 gram/day** | 35.1 | 42.5 | 27.2 | **Nondrinker** | 40.7 |
|  | >0.0-13.9 grams/day | 51.2 | 44.5 | 60.5 | < 1 drink/day | 30.7 |
|  | 14.0-27.9 grams/day | 8.2 | 8.2 | 8.4 | 1-<2 drinks/day | 8.1 |
|  | ≥28 grams/day | 5.5 | 4.8 | 3.9 | ≥2 drinks/day | 13.6 |
| Body mass index |  |  |  |  |  |  |
|  | **< 25 kg/m^2^** | 57.4 | 47.1 | 53.4 | **< 25 kg/m^2^** | 28.7 |
|  | 25-29.9 kg/m^2^ | 28.5 | 32.6 | 31.7 | 25-29.9 kg/m^2^ | 29.6 |
|  | ≥30 kg/m^2^ | 14.1 | 20.3 | 15.0 | ≥30 kg/m^2^ | 40.1 |
| Fruit and vegetable intake^g^ |  |  |  |  |  |  |
|  | 0-<2 servings/day | 8.5 | 9.9 | 5.8 | 0-199 grams/day | 49.2 |
|  | 2-<5 servings/day | 51.3 | 52.3 | 54.5 | 200-399 grams/day | 41.3 |
|  | **≥5 servings/day** | 40.3 | 37.8 | 39.7 | **≥400 grams/day** | 9.4 |
| Physical activity |  |  |  |  |  |  |
|  | <4 MET-hrs^h^/week | 34.1 | 27.9 | 17.3 | <4.2 MET-hrs/week | 57.9 |
|  | 4-<12.5 MET-hrs/week | 30.2 | 27.3 | 33.9 | 4.2-<12.5 MET-hrs/week | 15.6 |
|  | 12.5-<18 MET-hrs/week | 9.6 | 11.4 | 15.2 | 12.5-<16.7 MET-hrs/week | 5.4 |
|  | **≥18 MET-hrs/week** | 26.0 | 33.4 | 33.6 | **≥16.7 MET-hrs/week** | 21.1 |
| ^a^ Theoretical minimum risk (reference level) in bold | | | | | | |
| ^b^ Risk factors were selected based on the 2018 World Cancer Research Fund and American Institute for Cancer Research (WCRF/AICR) cancer prevention recommendations[21] for which the evidence for an association with breast cancer was considered convincing, probable, or suggestive  ^c^ US national exposure distributions for alcohol consumption were obtained from the National Health Interview Survey (NHIS 2013 and 2014), while information on BMI, fruit and vegetable intake and physical activity were obtained from the National Health and Nutrition Examination Survey (NHANES 2011-2012, 2013-2014). | | | | | | |
| ^d^ The measurement from the most recent questionnaire prior to breast cancer diagnosis, death, lost to follow-up or administrative end of follow-up for each questionnaire return cycle. | | | | | | |
| ^e^ The average of all past measurements for each follow-up cycle.  ^f^ The equivalent cut points for low- and high-risk groups, respectively, from the US prevalence data were: alcohol consumption (>0 vs 0 gram/day), BMI (≥25 vs <25 kg/m^2^), fruit and vegetable intake (<400 vs ≥400 grams/day), physical activity (<1000 vs ≥1000 MET-min/week).  ^g^ Sample average weight of fruits and vegetables: one apple (80g), 6 strawberries (100g), one orange (120g); 10 medium lettuce leaves (75g), 5 broccoli florets (100g), one medium carrot (60g). | | | | | | |
| ^h^ MET, metabolic equivalent of task, a measure of relative intensity of different physical activities as compared to the resting metabolic rate. | | | | | | |

| **Table S7. Age-standardized incidence rates of postmenopausal breast cancer in the low-risk group ^a^ and the overall Nurses' Health Study (NHS) population, and the US population, and the corresponding estimated population attributable risk (PAR%) according to the timing of the exposure measurements** | | | | | |
| --- | --- | --- | --- | --- | --- |
|  |  |  |  |  |  |
| Timing of the exposure measurements ^b^ | Incidence rate in the low-risk group in NHS  (per 100,000 person-year) | Incidence rate in NHS  (per 100,000 person-year) | Incidence rate in the US population (per 100,000 person-year) | PAR%  (NHS) | PAR%  (US population) |
| Baseline | 312.7 (260.5, 375.5) | 378.8 (369.4, 388.5) | 358.2 | 17.4% | 12.7% |
| Simple update | 283.5 (239.2, 336.0) | 378.8 (369.4, 388.5) |  | 25.2% | 20.8% |
| Cumulative average | 267.9 (218.9, 327.9) | 378.8 (369.4, 388.5) |  | 29.3% | 25.2% |
|  |  |  |  |  |  |

^a^ Low risk status defined by meeting all four criteria of alcohol consumption (0g/day), body mass index (<25kg/m2), fruit and vegetable intake (≥5 servings/day), and physical activity (≥18 total MET-hr/week).

^b^ (1) Baseline: using measurements of the exposures at study enrollment in 1986 (or 1990 for women who did not complete the 1986 FFQ); (2) Simple update: using the measurement of the exposures from the assessment just prior to diagnosis, death, lost to follow-up or administrative end; and (3) Cumulative average: using average exposure calculated from the assessment just prior to diagnosis, death, lost to follow-up or administrative end and all prior questionnaire cycles.
